# Identification of a Four-Gene Signature With Prognostic Significance in Endometrial Cancer Using Weighted-Gene Correlation Network Analysis

**DOI:** 10.3389/fgene.2021.678780

**Published:** 2021-09-20

**Authors:** Shijin Huang, Lihong Pang, Changqiang Wei

**Affiliations:** Department of Obstetrics and Gynecology, The First Affiliated Hospital of Guangxi Medical University, Nanning, China

**Keywords:** endometrial cancer, endometrial hyperplasia, prognostic biomarker, survival analysis, co-expression analysis, nomogram, WGCNA

## Abstract

Endometrial hyperplasia (EH) is a precursor for endometrial cancer (EC). However, biomarkers for the progression from EH to EC and standard prognostic biomarkers for EC have not been identified. In this study, we aimed to identify key genes with prognostic significance for the progression from EH to EC. Weighted-gene correlation network analysis (WGCNA) was used to identify hub genes utilizing microarray data (GSE106191) downloaded from the Gene Expression Omnibus database. Differentially expressed genes (DEGs) were identified from the Uterine Corpus Endometrial Carcinoma (UCEC) dataset of The Cancer Genome Atlas database. The Limma-Voom R package was applied to detect differentially expressed genes (DEGs; mRNAs) between cancer and normal samples. Genes with |log2 (fold change [FC])| > 1.0 and *p* < 0.05 were considered as DEGs. Univariate and multivariate Cox regression and survival analyses were performed to identify potential prognostic genes using hub genes overlapping in the two datasets. All analyses were conducted using R Bioconductor and related packages. Through WGCNA and overlapping genes in hub modules with DEGs in the UCEC dataset, we identified 42 hub genes. The results of the univariate and multivariate Cox regression analyses revealed that four hub genes, *BUB1B*, *NDC80, TPX2*, and *TTK*, were independently associated with the prognosis of EC (Hazard ratio [95% confidence interval]: 0.591 [0.382–0.912], *p* = 0.017; 0.605 [0.371–0.986], *p* = 0.044; 1.678 [1.132–2.488], *p* = 0.01; 2.428 [1.372–4.29], *p* = 0.02, respectively). A nomogram was established with a risk score calculated using the four genes’ coefficients in the multivariate analysis, and tumor grade and stage had a favorable predictive value for the prognosis of EC. The survival analysis showed that the high-risk group had an unfavorable prognosis compared with the low-risk group (*p* < 0.0001). The receiver operating characteristic curves also indicated that the risk model had a potential predictive value of prognosis with area under the curve 0.807 at 2 years, 0.783 at 3 years, and 0.786 at 5 years. We established a four-gene signature with prognostic significance in EC using WGCNA and established a nomogram to predict the prognosis of EC.

## Introduction

Endometrial cancer (EC) is one of the most common gynecological cancers worldwide, and its incidence has been increasing in developed countries in recent years (Siegel et al., 2019). The histological types of EC are divided into type I and II tumors (Morice et al., 2016; Passarello et al., 2019). Endometrioid adenocarcinoma (EAC) is the main histological subtype, accounting for nearly 70–80% of EC cases, and is known as an estrogen-dependent tumor (Passarello et al., 2019). The mechanism of genesis of type I tumors is associated with unopposed estrogen without progesterone protection (Brooks et al., 2019). Therefore, obesity, diabetes, and tamoxifen administration are the main risk factors for EC (Raglan et al., 2019; Njoku et al., 2020). Type I tumors often show an early onset symptom of vaginal bleeding, making them detectable at an early stage and thus are associated with a good prognosis (Brooks et al., 2019; Passarello et al., 2019). Conversely, type II tumors are less infrequent and are not associated with unopposed estrogen. Unlike type I tumors, late onset with late stage and undifferentiated histological types are common in type II tumors, whose pathologic subtype includes endometrial serous carcinoma, clear-cell carcinoma, and carcinosarcoma; as a result, they are associated with an unfavorable prognosis (Siegel et al., 2019). Thus, the early diagnosis of EC is essential for changing the outcome of EC treatment. However, there is still no effective screening method for EC, especially in postmenopausal women without symptoms (Passarello et al., 2019).

The long-term effect of unopposed estrogen on the endometrium results in endometrial hyperplasia (EH), which is the precursor for EC, and its incidence is considerably greater than that of EC in premenopausal women (Sanderson et al., 2017). According to the 2014 guidelines, the pathological classifications of EH are non-atypical EH (benign hyperplasia) and atypical EH or endometrial intraepithelial neoplasia (EIN)/well-differentiated carcinoma (Emons et al., 2015). If left untreated, EH gradually develops into EC (Doherty et al., 2020). Thus, the early diagnosis and treatment of EH have a great effect on the prevention of EC. Dilatation and curettage are the only traditional procedures used to obtain endometrial tissue from women with abnormal vaginal bleeding (Sanderson et al., 2017). Thus, similar to the case of EC, there are no less-invasive methods for the diagnosis and screening of EH.

Nearly one-third of women with atypical hyperplasia have concurrent EC, and the risk of progression from atypical hyperplasia to EC is 8% per year (Doherty et al., 2020). The pathogenesis of EH in EC, which involves complicated molecular mechanisms, has not been fully elucidated (Sanderson et al., 2017). Moreover, there is still no strong biomarker for the early diagnosis of EH or EC. The human genome has been employed to investigate various diseases with considerable progress (Gonzaga-Jauregui et al., 2012). Thus, it is important to determine whether there are crucial genes involved in the progression of EH to EC.

The application of microarray data or RNA sequencing (RNA-Seq) data in tumor genome sequencing has enabled the discovery of genomic biomarkers leading to cancer diagnosis and prognosis, and the differences between microarray data or RNA-Seq data have been discussed elsewhere (Febbo and Kantoff, 2006; Damodaran et al., 2015; Robinson et al., 2015). Therefore, to identify key genes with prognostic significance for the progression of EH to EC, a comprehensive bioinformatic analysis was performed in this study using weighted-gene correlation network analysis (WGCNA) (Langfelder and Horvath, 2008), which is a valid method for searching hub genes as proved by several studies (Das et al., 2017; Yin et al., 2018; Li et al., 2019; Yao et al., 2019; Zhu et al., 2019). In this study, we utilized microarray data and RNA-Seq data to identify a robust prognostic gene signature that may be directly incorporated into a clinical practice for prognostic prediction.

## Materials and Methods

### Data Sources and Processing

**FIGURE 1:**
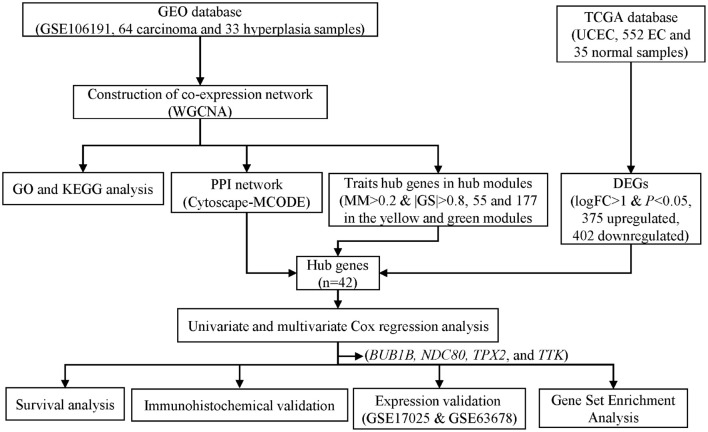
Flowchart of screening hub genes and validation. GEO, Gene Expression Omnibus. TCGA, the Cancer Genome Atlas. GO, Gene ontology. KEGG, Kyoto Encyclopedia of Genes and Genomes. UCEC, uterine corpus endometrial carcinoma. PPI, protein-protein interaction. MCODE, Molecular Complex Detection. MM, module membership. GS, gene significance. DEG, differentially expressed genes.

To obtain microarray data of EC and EH, we performed a comprehensive search of the Gene Expression Omnibus (GEO) database^1^ with keywords “endometrial cancer” and “endometrial hyperplasia.” Datasets with mRNA expression profiles were included. Finally, dataset GSE106191 was included in the study. The study design is shown in Figure 1. The mRNA expression profiles in dataset GSE106191, which consists of data of 64 carcinoma samples and 33 hyperplasia samples based on the platform of GPL570 (Affymetrix Human Genome U133 Plus 2.0 Array), were downloaded from the GEO. The dataset was designed for expression microarray experiments of mRNA from endometrial cancer tissue and endometrial hyperplasia. The main characteristics of the samples are listed in Table 1. The median age (50 years old) of the patients was calculated and the samples were divided into two groups according to the median age (younger and older groups, respectively). The expression data were processed with background correction and normalization using the limma R package (Supplementary Figures 1A,B). Principal component analysis (PCA) plots and heatmaps are shown in Supplementary Figures 1C,D.

**TABLE 1:** The characteristics of samples of GSE106191.

Variate	Tissue	*N*	Range	Mean	Standard deviation	*p*
Age (years old)	Endometrial cancer	64	27.5–77.5	51.953yr.	11.343	
	Hyperplasia	33	27.5–72.5	47.803yr.	9.917	0.211

RNA-Seq data of gene expression with log2(x + 1) transformation in uterine corpus endometrial carcinoma (UCEC) datasets, which consist of 552 EC samples and 35 normal samples from the Cancer Genome Atlas (TCGA) database, were downloaded from UCSC Xena^2^. Related clinical information of the cancer samples was also downloaded for survival analysis. After background correction and normalization, the limma-voom R package was applied to detect differentially expressed genes (DEGs; mRNAs) between cancer and normal samples (Law et al., 2014; Ritchie et al., 2015). Genes with |log2 (fold change [FC])| > 1.0 and *p* < 0.05 were considered as DEGs.

### Construction of the Weighted-Gene Correlation Network Analysis Network

For understanding the interrelationship among the selected genes and for identifying gene modules and key genes responsible for a particular clinical trait, we constructed a co-expression network using the “WGCNA” package in R Studio (Langfelder and Horvath, 2008) with the top 75% genes (*n* = 8813) with a maximal significant median absolute deviation (MAD) using the samples in GSE106191. The clinical traits, including subtype, age, and related information, are summarized in Table 1. First, we constructed a hierarchical clustering tree using an expression matrix to detect outliers. We then calculated the scale-free topology fit index as a function of the soft-thresholding power according to the network topology analysis function. Next, the intramodular connectivity between genes with similar expression profiles was calculated using the topological overlap dissimilarity measure (TOM). These genes were divided into different modules with a minimum of 30 genes per module. Hierarchical clustering and dynamic tree cut were used to identify modules, and module eigengene (ME, defined as the first principal component of a given module and can be considered a representative of the gene expression profiles in a module) networks were utilized to study the module relationship. Intramodular connectivity and module membership (MM) were screened for intramodular hub genes and calculated to identify genes with a high gene significance (GS), which represented the correlation between genes and samples, as well as high intramodular connectivity in interesting modules. Trait-hub genes, defined as |MM| > 0.8 and |GS| > 0.2, were exported for further analysis in the module of interest.

The R package “clusterProfiler” was used to conduct Gene Ontology (GO) and Kyoto Encyclopedia of Genes and Genomes (KEGG) enrichment analyses of genes and hub genes in each module, and results with *p* < 0.05 were considered statistically significant.

### Integration of the Protein–Protein Interaction (PPI) Network and Identification of Hub Genes

To visualize the integrated regulatory networks with connectivity between genes, we exported the corresponding topological overlap in the module with a significant module-trait-correlation and *p* value using Cytoscape software (version 3.7.2) (Shannon et al., 2003). We then employed the MCODE application (Bader and Hogue, 2003) plug-in in Cytoscape to detect clusters with the closest connectivity at a degree cut-off and k-core of 2. The genes detected in the MCODE cluster were considered hub genes and were subsequently exported to overlap with DEGs from the UCEC dataset.

### Univariate and Multi-Cox Regression Analyses

To screen hub genes that were significantly associated with the overall survival (OS) of EC, we performed a univariate Cox proportional hazards regression analysis. Genes with *p* < 0.05 were considered prognostic genes of EC. We performed survival analysis using the hub genes with the “survival package” and “survminer package.” Kaplan–Meier survival curves were plotted according to the expression profiles from the UCEC dataset, which were divided into two groups based on an optimal expression cutoff for genes as determined using survminer. In the univariate Cox proportional hazards regression analysis, genes with *p* < 0.05 were pooled for multivariable Cox regression model construction with stepwise regression.

We also carried out multivariate Cox regression analysis with stepwise regression to construct the proportional hazard model with major clinical factors of patients from the TCGA dataset. A linear combination of hub genes weighted by their regression coefficients derived from the multivariate Cox regression model was applied to predict a risk score that was employed as a marker for receiver operating characteristic (ROC) curves. Time-dependent ROCs of the risk score with a comparison of sensitivity and specificity of the survival rate were analyzed using the “timeROC” package and visualized using the “ggplot2” R package. We applied Benjamini–Hochberg false discovery rate (FDR, with threshold < 0.05) multiple hypothesis correction when indicated. We then tested proportional hazards assumption with the cox.zph function in the “survival package” for each model.

### Validation of Hub Gene Expression and Pathology

For the validation of hub gene expression, we compared the expression of hub genes between normal hyperplasia and tumor tissues. The GSE17025 and GSE63678 datasets from GEO were also downloaded for the validation of expression. Pathological and immunohistochemical staining data were acquired from the Human Protein Atlas (HPA^3^).

### Gene Set Enrichment Analysis (GSEA)

To identify potential pathways of the hub genes, we performed a gene set enrichment analysis (GSEA). According to the risk scores of the hub genes, the UCEC tumor samples were divided into high- and low-risk groups. The GSEA was used to identify potential pathways based on the gene sets ‘‘h.all.v7.2.symbols’’ downloaded from the GSEA homepage^4^.

### Statistical Analysis

All statistical analyses were performed using R programming language (version 4.0.2) and Bioconductor (version 3.2). Statistical significance was set at *p* < 0.05.

## Results

### Identification of Differentially Expressed Genes in the Cancer Genome Atlas-Uterine Corpus Endometrial Carcinoma Dataset

A total of 15,023 genes with an FDR cutoff <0.05 was applied to the differential expression analysis with limma. We found that 375 and 402 DEGs were upregulated and downregulated, respectively, in 552 EC samples and 35 normal samples from the UCEC dataset. The heatmap, PCA plots, and volcano plots are shown in Supplementary Figures 2A–C. The characteristics of patients who were divided into two groups according to survival status are shown in Table 2. Finally, we included 543 samples with enough survival information. There was a significant difference between the groups in terms of age, FIGO stage, histological type, and grade (Table 2).

**TABLE 2:** The characteristics of samples in TCGA-UCEC dataset.

Variant	Dead	Alive	*p*	SMD	Missing
**N**	91	452			
**All samples**					
EC (*n* = 543)	91	452			
Normal (*n* = 35)	–	–			
**Age (%)**		0.005		0.36	0.6
<60	18 (19.8)	160 (35.6)			
> = 60	73 (80.2)	289 (64.4)			
**Menopause_ status (%)**			0.323	0.251	5.3
Indeterminate	1 (1.1)	16 (3.8)			
Perimenopause	1 (1.1)	16 (3.8)			
Postmenopause	81 (90.0)	364 (85.8)			
Premenopause	7 (7.8)	28 (6.6)			
**BMI (%)**			0.808	0.076	5.7
Normal	18 (20.7)	77 (18.1)			
Obesity	49 (56.3)	254 (59.8)			
Overweight	20 (23.0)	94 (22.1)			
**Race (%)**			0.896	0.128	5.9
American India or Alaska native	1 (1.1)	3 (0.7)			
Asian	2 (2.3)	18 (4.3)			
Native Hawaiian or other Pacific islander	19 (21.3)	87 (20.6)			
Black or African American	2 (2.3)	7 (1.7)			
White	65 (73.0)	307 (72.7)			
**Ethnicity (%)**			0.486	0.129	28.5
Hispanic or Latino	4 (6.2)	11 (3.4)			
Not Hispanic or Latino	61 (93.8)	312 (96.6)			
**Histological type (%)**			<0.001	0.559	0
EEC	49 (53.8)	358 (79.2)			
MSE	6 (6.6)	16 (3.5)			
SEA	36 (39.6)	78 (17.3)			
**Grade (%)**			<0.001	0.75	0
G1	2 (2.2)	96 (21.2)			
G2	14 (15.4)	106 (23.5)			
G3	69 (75.8)	245 (54.2)			
High Grade	6 (6.6)	5 (1.1)			
**FIGO Stage (%)**			<0.001	0.791	0
I	32 (35.1)	307 (67.9)			
II	9 (9.9)	42 (9.3)			
III	33 (36.3)	91 (20.1)			
IV	17 (18.7)	12 (2.7)			

*SMD, standardized mean difference; EC, endometrial cancer; BMI, body mass index; EEC, endometrioid endometrial adenocarcinoma; MSE, Mixed serous and endometrioid; SEA, serous endometrial adenocarcinoma; FIGO, Federation International of Gynecology and Obstetrics.*

### Construction of Weighted Co-expression Network

**FIGURE 2:**
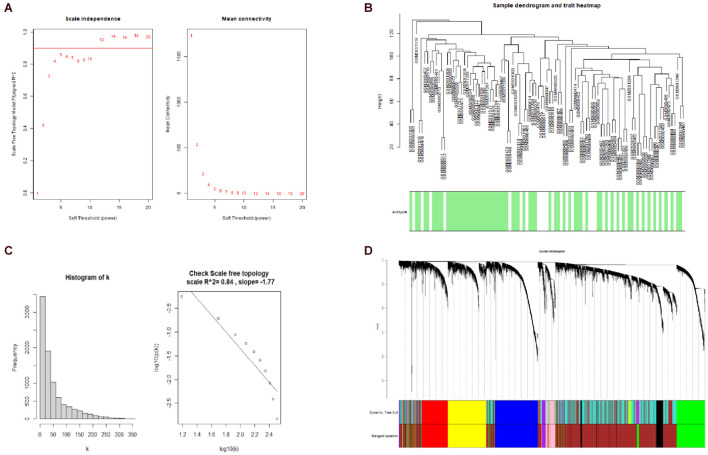
Weighted-gene correlation network analysis (WGCNA). **(A)** Analysis of the scale-free topology model fit index for soft threshold powers (β) and the mean connectivity for β. **(B)** Histogram of K and check scale-free topology. **(C)** Sample clustering for detecting outliers, and sample dendrogram and trait heatmap. **(D)** Cluster dendrogram, dynamic tree cut and merged dynamic color plot.

**FIGURE 3:**
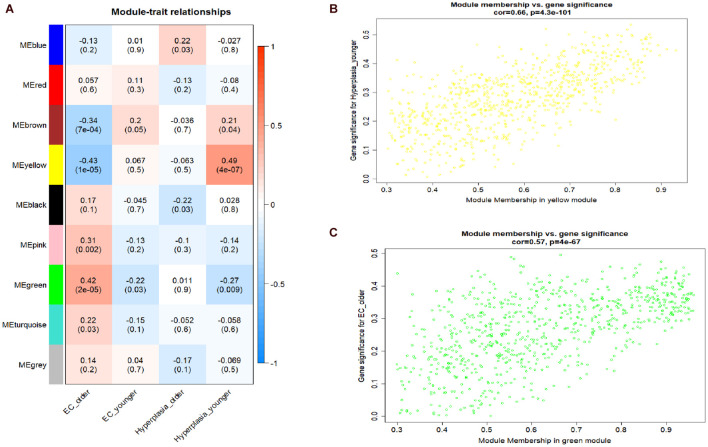
Module-trait relationships plot and scatterplot of gene significance vs. module membership. **(A)** Module-trait relationships plot. Each row represents a module eigengene, each column represents a clinical trait, and each cell consists of the corresponding correlation and *p* value, which are color-coded by correlation according to the color legend. **(B)** Scatterplot of gene significance (*y*-axis) vs. module membership (*x*-axis) in yellow modules. **(C)** Scatterplot of gene significance (*y*-axis) vs. module membership (*x*-axis) in yellow and green modules.

A hierarchical clustering tree established with the 8,813 genes of the samples in GSE106191 revealed no outliers, and thus no outlier was removed (Figure 2C). A β value of 5 (scale-free *R*^2^ = 0.85) was selected as the appropriate soft-threshold value to construct a scale-free network (Figures 2A,B). Finally, nine modules with an unsigned TOM type were identified (Figure 2D). Module and trait relationships were established, which indicated that genes in the yellow and green modules were significantly associated with EC and EH (Figure 3A). The yellow module positively correlated with the trait of hyperplasia in the samples from younger patients, with a correlation coefficient of 0.66 and *p* value of 4.3 × 10^–101^ (Figure 3B). Furthermore, the green module significantly correlated with the trait of EC in the samples from older patients, with a correlation coefficient of 0.57 and *p* value of 4 × 10^–67^ (Figure 3C). Other related heatmaps are shown in Supplementary Figure 3.

### Functional Enrichment Analysis

**FIGURE 4:**
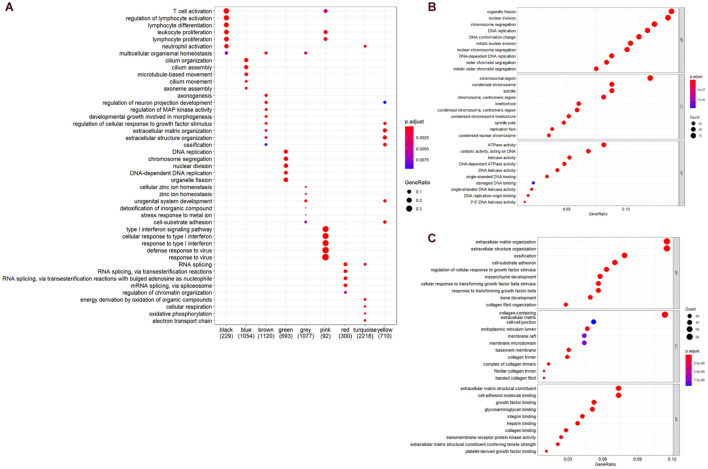
Gene ontology (GO) and pathway enrichment analysis. **(A)** GO analysis of all module genes (the top five categories were presented). **(B)** GO analysis of the genes in the yellow module (BP, biological process; CC, cellular component; MF, molecular function). **(C)** GO analysis of the genes in the green module.

**FIGURE 5:**
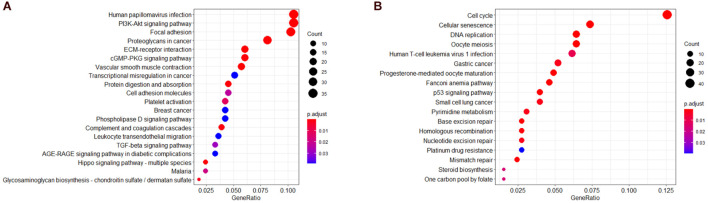
Kyoto Encyclopedia of Genes and Genomes (KEGG) pathway analysis. **(A)** KEGG pathway of genes in the yellow modules. **(B)** KEGG pathway of genes in the green modules.

To further understand the biological significance of the two modules and other modules, the GO (biological process, cellular component, and molecular function) (Figure 4) and KEGG pathway enrichment analyses were performed. The results showed that genes clustered in the yellow module were mainly involved in extracellular structure organization (GO:0006281) (Figure 4B) and were significantly enriched in the human papillomavirus infection (hsa05165) and PI3K-Akt signaling pathway (hsa04151) (Figures 5A,B), which is considered the main tumorigenic pathway for EC. Those clustered in the green module were mainly involved in organelle fission (GO:0048285) (Figure 4C) and were significantly enriched in the cell cycle and DNA replication (hsa04110 and hsa03030) (Figure 5B).

### Identification of the Hub Modules and Genes

According to the module and trait relationships (Figure 3A), the most important genes associated with EC and EH were clustered in the yellow and green modules. The high correlation between gene significance and module membership implies that hub genes in the yellow or green module tend to be highly correlated with EC or EH. This suggests that both gene significance and module membership (intramodular connectivity) can be combined in a systems biologic screening method for identifying EC or EH related genes (Figure 3). This is one of the methods applied in WGCNA to identify hub genes. In other modules, gene significance and module membership were less correlated with EC or EH. Moreover, the GO and KEGG pathway analyses both implied genes in the yellow and green modules were associated with EC. Therefore, priority was given to the yellow and green modules to identify hub genes.

**FIGURE 6:**
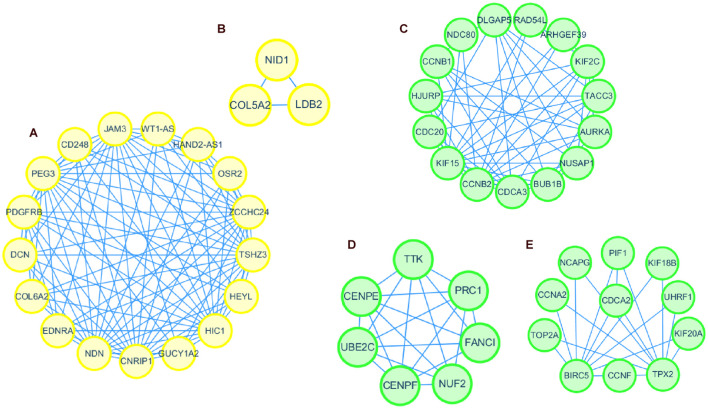
Protein interaction network of genes in clusters identified by MCODE in Cytoscape. Clusters 1 and 2 formed the yellow module **(A,B)**. Clusters 1, 2, and 3 formed the green module **(C–E)**.

**FIGURE 7:**
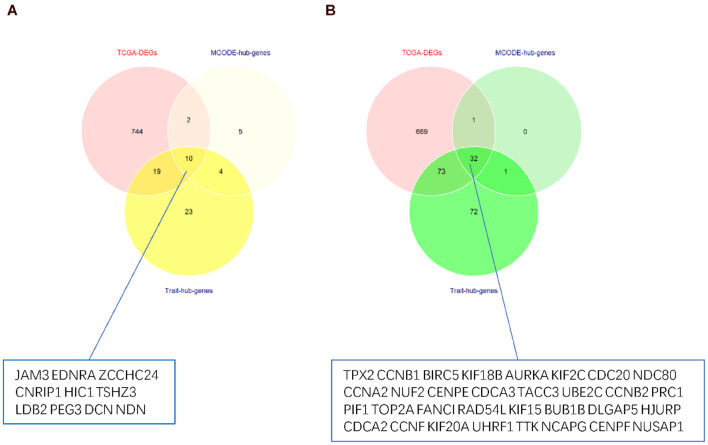
Venn diagram of overlapping genes from TCGA-DEGs, MCODE-hub genes, and Trait-hub genes. **(A)** Hub genes from the yellow module. **(B)** Hub genes from the green module.

Thus, the genes in these two modules were recalculated for topological overlap, and the corresponding overlaps with edges, nodes, and weighted values were exported. Thereafter, the top 500 weighted-value edges and nodes were selected for further analysis using Cytoscape software (version 3.7.2). Finally, two clusters with 20 nodes and 98 edges were found in the yellow module, and three clusters with 33 nodes and 91 edges were identified in the green module through the MCODE application (Supplementary Table 1; Figure 6). Trait-hub genes (56 and 178 in the yellow and green modules, respectively) in these two modules were also exported. The genes in the MCODE clusters and trait-hub genes were overlapped with the DEGs in the UCEC dataset. In all, 10 hub genes from the yellow module, which were all downregulated, and 32 hub genes from the green module, all of which were upregulated DEGs, were identified after overlapping (Figures 7A,B). Overlapping genes were exported for survival analysis to screen potential prognostic factors.

### Survival Analysis

**FIGURE 8:**
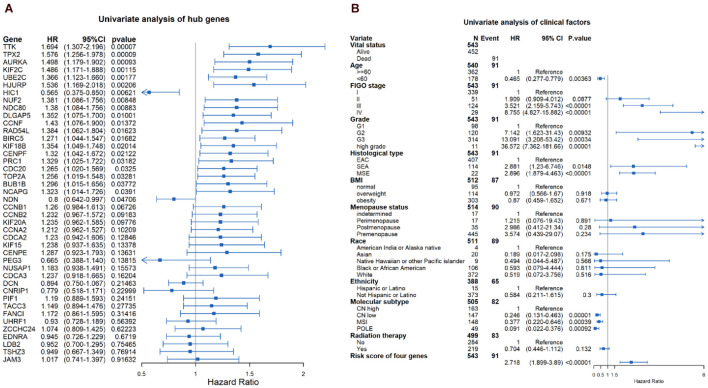
Univariate analysis of the hub genes **(A)** and clinical factors **(B)** in the UCEC dataset.

**FIGURE 9:**
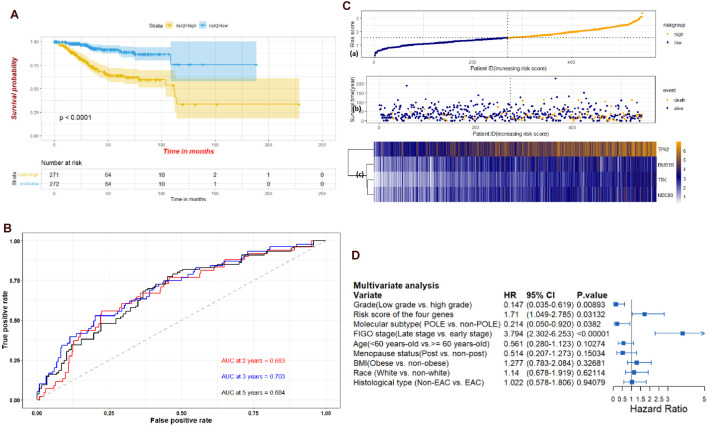
**(A)** Kaplan-Meier plots of the UCEC dataset stratified by high-risk and low-risk groups based on median risk score of the four genes. **(B)** Time-dependent ROC plot risk scores of the four hub genes. **(C)** The risk score performance of the four hub genes in the UCEC dataset. **(D)** Multivariate analysis of clinical factors in the UCEC dataset.

Through the univariate Cox-regression analysis, 21 of the 42 hub genes were found to be associated with the prognosis of EC, namely, *TTK*, *TPX2*, *AURKA*, *KIF2C*, *UBE2C*, *HJURP*, *HIC1*, *NUF2*, *NDC80*, *DLGAP5*, *CCNF*, *RAD54L*, *BIRC5*, *KIF18B*, *CENPF*, *PRC1*, *CDC20*, *TOP2A*, *BUB1B*, *NCAPG*, and *NDN* (Figure 8A). For the UCEC dataset, the univariate analysis revealed that age < 60 years, POLE (DNA polymerase epsilon), low copy number (CN), and microsatellite instability (MSI) of the molecular subtype had a favorable prognosis for EC. In contrast, mixed serous and endometrioid, serous endometrial adenocarcinoma of histological type, FIGO stages III–IV, grades 2–3, high grade, and risk score of four genes were associated with a poor prognosis (Figure 8B). The results of the multivariate Cox-regression analysis with stepwise regression indicated that BUB1B (mitotic checkpoint serine/threonine kinase B), NDC80 (NDC80 kinetochore complex component), TPX2 (microtubule nucleation factor), and TTK (TTK protein kinase) correlated with the prognosis of EC (Table 3). The results of the Kaplan–Meier survival analysis also showed that patients with a lower expression of the four genes had better OS (Supplementary Figure 4) compared with a higher expression. The AUCs for BUB1B, NDC80, TPX2, and TTK for the prediction of OS were 0.569, 0.594, 0.637, and 0.627, respectively. The risk score was calculated as follows: 0.8871 × expression of TTK + (−0.5266 × expression of BUB1B) + (−0.5022 × expression of NDC80) + 0.5177 × expression of TPX2 (Table 3). According to the median risk score, patients with EC were divided into high- and low-risk groups. The results of the Kaplan–Meier survival analysis indicated that high-risk patients had poor OS (Figure 9A). The AUCs with respect to the ROCs for 2-, 3-, and 5-year OS were 0.683, 0.703, and 0.684, respectively (Figure 9B). The distributions of the risk scores, survival status, and survival duration of the 543 EC patients and the expression heatmap for the 4 genes are shown in Figure 9C. For the clinical factors in the UCEC dataset, age group, grade, tumor stage, and molecular subtype were independently associated with the prognosis of EC (Figure 9D).

**TABLE 3:** Multivariant analysis of hub genes.

Genes	Coefficients	HR	95%CI	*p*
NDC80	−0.502	0.605	0.3715–0.986	0.044
BUB1B	−0.527	0.591	0.3827–0.912	0.017
TTK	0.887	2.428	1.372–4.297	0.002
TPX2	0.518	1.678	1.132–2.488	0.010

*HR, hazard ratio; CI, confidence interval.*

**FIGURE 10:**
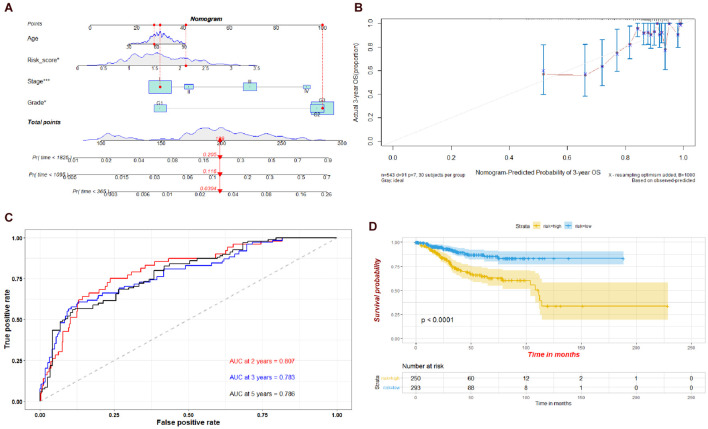
**(A)** Nomogram of the risk scores of the four hub genes, age, stage, and grade. **(B)** Calibration curve for the nomogram. **(C)** Time-dependent ROC plot of the risk model used in the nomogram. **(D)** Kaplan-Meier plots of the risk model used in the nomogram.

To evaluate the prognostic value of the risk model established by the four genes, we established a nomogram integrating the risk score, age, tumor stage, and grade. Calibration plots showed that the actual 3-year OS was consistent with the nomogram-predicted probability of 3-year OS (Figures 10A,B). The ROC plot also indicated that the risk model had a potential predictive value for prognosis, with an AUC of 0.807 at 2 years, 0.783 at 3 years, and 0.786 at 5 years (Figure 10C). The results of the Kaplan–Meier survival analysis suggested that the high-risk group had a poorer prognosis than the low-risk group (Figure 10D).

Meanwhile, to validate the risk score value, we divided the UCEC dataset into training and testing sets randomly in a 1:1 ratio. The ROC curves and the results of the Kaplan–Meier survival analysis in the training and testing sets were consistent with the results of the entire dataset (Supplementary Figures 5A–D). Both univariate and multivariate analyses with the training and testing sets showed that the risk score of the four genes could be an independent prognostic factor for EC (Supplementary Table 2). The AUCs for the risk score, age, stage, and grade for the prediction of OS were 0.669, 0.513 0.649, and 0.692, respectively (Supplementary Figure 5E). In addition, the correlation analysis showed that there was a strong correlation between the four genes (Supplementary Figure 5F).

### Gene Set Enrichment Analysis

**FIGURE 11:**
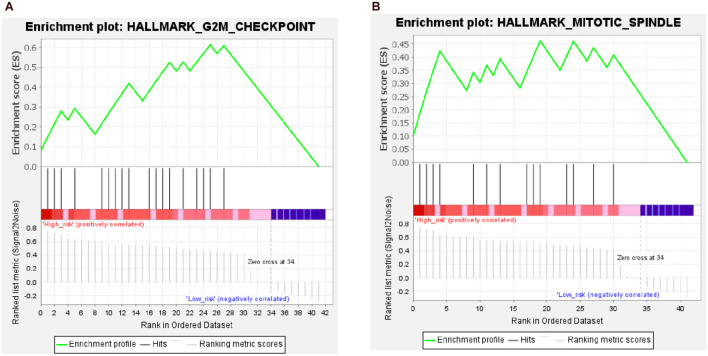
Results from gene set enrichment analysis (GSEA) of hub genes in the high-risk group. **(A)** Hallmark G2M checkpoint pathway. **(B)** Hallmark mitotic spindle pathway.

The results of the GSEA showed that two gene sets were significant at FDR < 25% in the high-risk group; these were involved in the G2M checkpoint pathway and mitotic spindle of the cell cycle (Figure 11 and Supplementary Table 3). However, none of the gene sets was enriched in the low-risk group.

### Validation of Expression and Immunohistochemistry (IHC) of Hub Genes

**FIGURE 12:**
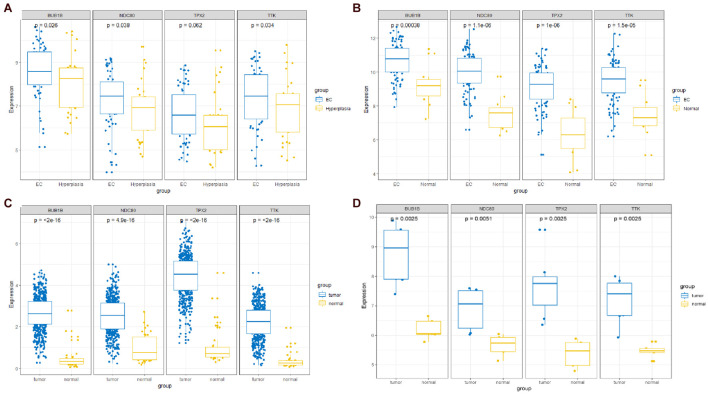
Comparisons of the four-hub gene expression in different datasets between endometrial cancer (EC) and endometrial hyperplasia (EH) or normal tissue. **(A)** GSE106191 dataset. **(B)** GSE17025 dataset. **(C)** UCEC dataset. **(D)** GSE63678 dataset.

**FIGURE 13:**
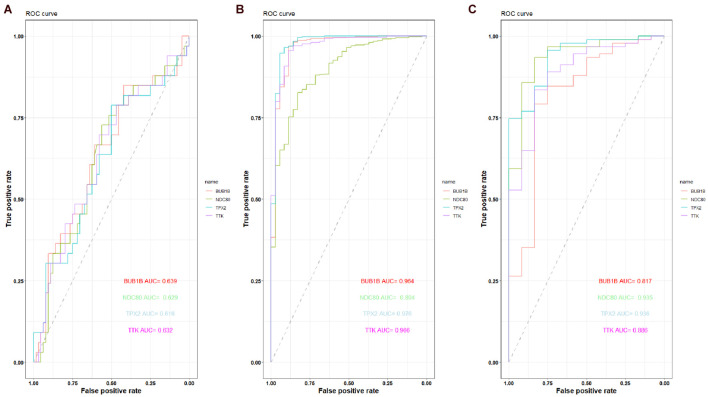
Receiver operating characteristic (ROC) curve of the capacity of BUB1B, NDC80, TPX2, and TTK in distinguishing between EC, EH, or normal tissue. **(A)** ROC curve of the capacity of BUB1B, NDC80, TPX2, and TTK in distinguishing between EC and EH in the GSE106191 dataset. **(B)** ROC curve of the capacity of BUB1B, NDC80, TPX2, and TTK in distinguishing between EC and normal tissue in the UCEC dataset. **(C)** ROC curve of the capacity of BUB1B, NDC80, TPX2, and TTK in distinguishing between EC and normal tissue in the GSE17025 dataset.

The expression of the four hub genes was upregulated in the EC samples. The expression of the four genes in the entire UCEC dataset, training set, and testing set in tumor samples was higher than that in the normal samples (Supplementary Figure 6). There was no significant difference in the expression of TPX2 between the EC and EH samples in the GSE106191 dataset (Figure 12A). However, in the GSE17025, UCEC, and GSE63678 datasets, all four genes were significantly differentially expressed between the normal and tumor samples (Figures 12B–D). The ROC curves indicated that the four genes had a strong ability to distinguish tumor tissues from EH or normal tissues (Figure 13).

**FIGURE 14:**
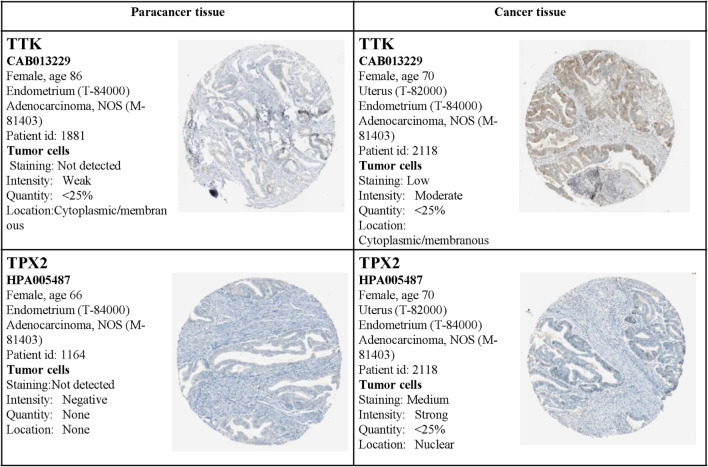
Validation of the four hub genes by the Human Protein Atlas (HPA) database. Data for BUB1Band NDC80 were not available in the HPA database.

Moreover, the expression status of the hub genes determined by IHC was acquired from the Human Protein Atlas database (see text footnote 3). The results also demonstrated that the expression levels were in accordance with the transcription level, but related IHC data for BUB1Band NDC80 were not available from the database (Figure 14).

## Discussion

Although the incidence of EC is increasing, most patients diagnosed with type I EC at an early stage have a good prognosis. Age, tumor histological type, stage, and tumor grade are the main independent prognostic factors of EC (Lewin, 2011; Morice et al., 2016). Depth of myometrial invasion and lymphovascular space invasion is the main predictor of prognosis after surgery (Brooks et al., 2019). During the past decade, considerable progress has been made in understanding the complex molecular and cellular nature of EC, and various key genes have been identified through bioinformatic analyses (Huo et al., 2019; Bian et al., 2020; Liu et al., 2020). Through WGCNA, we determined the most significant modules using clinical traits. We selected hub genes with a high degree of gene clustering as well as GS and MM, in which the relationship between clinical traits and modules was the most significant. The present study showed that genes associated with EC and EH were mainly clustered in the green and yellow modules. The enriched functional GO terms and KEGG pathways confirmed these genes in these two modules. Moreover, there was a significant difference in expression between normal and EC samples in the TCGA dataset and the validation dataset of GSE17025 and GSE63678.

Through comprehensive bioinformatic analysis, we identified 42 hub genes, of which 21 genes were found to be associated with the prognosis of EC. Moreover, in the multivariate model, a high expression of TTK and TPX2 was associated with a poor prognosis of EC, but BUB1B and NDC80 exhibited a strong prognostic value for EC, which indicated that these four genes have a robust and independent prognostic value for EC. The results for the multivariate Cox-regression analysis of BUB1B and NDC80 were opposite to the results for the univariate Cox-regression analysis. This is because each gene was assessed through separate univariate Cox regressions. However, in the multivariate regression analysis, all 21 genes were assessed together and the confounding genes on BUB1B and NDC80 were considered. The nomogram constructed using the risk score of the four genes with age, tumor stage, and grade had a strong ability to predict EC prognosis, which suggested that the four genes could be used as biomarkers for clinical application.

In 2013, the TCGA consortium first classified the integrated genomic characterization of EC into four categories, from the best prognosis to the worst as follows: POLE ultramutated, microsatellite instability hypermutated (MSI-H), copy number low (CNL), and copy number high (CNH) (Cancer Genome Atlas Research Network et al., 2013). These molecular subtyping systems, which were based on the assessment of 363 endometrial carcinomas, enable a better understanding of the tumorigenesis of EC and improve treatment decisions and outcomes. Here, more samples were added to the UCEC dataset. Our analysis based on 543 ECs showed that patients with POLE ultramutated had a favorable prognosis compared with those with the other molecular subtypes.

BUB1B is a kinase involved in spindle checkpoint function and plays an important role in inhibiting anaphase-promoting complex/cyclosome (APC/C), delaying the onset of anaphase, and ensuring proper chromosome segregation (Abal et al., 2007). Bioinformatics analyses have revealed that the overexpression of BUB1B is associated with an unfavorable prognosis in liver cancer, pancreatic cancer, and lung cancer based on the TCGA database (Dong et al., 2019; Yang et al., 2019; Dai et al., 2020). In this study, we determined that a high expression of BUB1B is associated with a poor prognosis of EC. These findings indicate that it may be a potential biomarker for predicting the prognosis of EC. The overexpression of BUB1B may be involved in impaired spindle checkpoint function, which is linked to different cancers. However, the mechanism underlying its role in the prognosis of EC is yet to be investigated.

NDC80, which is a component of the essential kinetochore-associated NDC80 complex and is required for chromosome segregation and spindle checkpoint activity, was observed to inhibit cancer cell proliferation and induce apoptosis after knockdown (Qu et al., 2014; Liu et al., 2019; Ustinov et al., 2020). Xing et al. (2016) found that NDC80 promotes the proliferation and metastasis of colon cancer cells by promoting aneuploidy. Moreover, silencing NDC80 expression suppressed the proliferation and cell cycle progression of pancreatic cancer lines (Meng et al., 2015). These results further support the hypothesis that NDC80 is potentially involved in the genesis of EC. However, the mechanism of action of NDC80 in EC genesis requires further investigation.

*TPX2* is known as a cancer-related gene that is involved in the normal assembly of mitotic spindles and microtubules during apoptosis and is also associated with unfavorable prognosis in lung cancer, liver cancer, pancreatic cancer, and renal cancer (Kadara et al., 2009; Asteriti et al., 2010; Neumayer et al., 2014). A recent study (Jiang et al., 2018) established that the silencing of TPX2 expression positively associates with the inhibition of EC development. TPX2 can feature prominently in the oncogenic potential of Aurora A, which is a key regulator of mitosis (Asteriti et al., 2010). Similarly, its importance in the cell cycle helps understand the association of its overexpression with an unfavorable prognosis in EC. However, its role in the genesis of ECs is unknown and needs to be explored. Surprisingly, no differences were observed in expression between EC and EH samples in the GSE106191 dataset. Conversely, the expression of TPX2 in the UCEC and GSE17025 datasets was significantly higher in the EC samples than in the normal samples. Thus, it is possible that TPX2 may be overexpressed during EH formation. However, more data are needed to support this assumption.

TTK protein kinase, also called CT96 or MPS1, which phosphorylates proteins at serine, threonine, and tyrosine, may be associated with cell proliferation and could be essential for chromosome alignment by enhancing the AURKB activity (via direct CDCA8 phosphorylation) at the centromere as well as the mitotic checkpoint. Previous studies have revealed that a high expression of TTK is associated with a poor prognosis for EC, liver cancer, and pancreatic cancer (Kaistha et al., 2014; Liu et al., 2015; Yang et al., 2020). It has been reported that MPS1 was predominantly expressed in human breast tumors and activated by the loss of function mutation of TP53, which is also known as the main determinant of tumorigenesis in EC (Győrffy et al., 2014). However, no study has confirmed the relationship between TTK and EC prognosis.

Overall, BUB1B, NDC80, TPX2, TTK, and other hub genes identified in this study were all involved in the cell cycle, which was consistent with the GSEA results. Increasing evidence has shown that the cell cycle plays an important role in cancer progression (Wiman and Zhivotovsky, 2017). Each stage of the cell cycle is regulated by precise molecular mechanisms. Any changes leading to the dysregulation of the cell cycle, including DNA damage and mutations or improper chromosomal segregation and aneuploidy, result in genomic instability, a distinct characteristic of cancer (Wenzel and Singh, 2018). These results further support the hypothesis that overexpression of these four genes might cause dysregulation of the endometrium cell cycle. Moreover, these four genes may be novel targets for the treatment of EC.

This study had some limitations. First, we did not export all genes for analysis in modules other than the yellow and green modules, which would have helped to identify more genes that are significantly associated with the prognosis of EC. Second, experimental validation of hub genes has not yet been performed because of time limitations. Third, no more data than what we used here regarding EH are publicly available to assess gene changes from EH to EC. Although we found four genes to be associated with EC prognosis, we did not have enough evidence to elaborate on the genes involved in the process of EH progression to EC.

## Conclusion

Through comprehensive analysis via WGCNA on datasets with EH and EC, we identified a four-gene signature with prognostic significance in EC. In addition, a nomogram established with risk scores for the four genes as well as age, tumor stage, and grade might have great value in predicting the OS of patients with EC. This study offers new insights into the molecular mechanisms of EC progression and prognosis. However, considerably more work must be done to determine biomarkers for the progression of EH to EC.

## Data Availability Statement

The raw data supporting the conclusions of this article will be made available by the authors, without undue reservation.

## Author Contributions

SH and LP contributed to the conception and design of the study. SH and CW organized the database and performed the statistical analysis. SH wrote the first draft of the manuscript and sections of the manuscript. All authors contributed to manuscript revision while also reading and approving the submitted version.

## Conflict of Interest

The authors declare that the research was conducted in the absence of any commercial or financial relationships that could be construed as a potential conflict of interest.

## Publisher’s Note

All claims expressed in this article are solely those of the authors and do not necessarily represent those of their affiliated organizations, or those of the publisher, the editors and the reviewers. Any product that may be evaluated in this article, or claim that may be made by its manufacturer, is not guaranteed or endorsed by the publisher.

## Footnotes

1
http://www.ncbi.nlm.nih.gov/gds/

2
https://gdc.xenahubs.net

3
https://www.proteinatlas.org/

4
http://www.gsea-msigdb.org/gsea/downloads.jsp
