# Supplementary material for: Identification of a Four-Gene Signature With Prognostic Significance in Endometrial Cancer Using Weighted-Gene Correlation Network Analysis
Source: Front Genet. 2021 Sep 20;12:678780. doi: 10.3389/fgene.2021.678780 (PMC8488359; doi:10.3389/fgene.2021.678780)
Supplement: Supplementary file 1 [file Data_Sheet_1.docx]

Supplementary Materials

# Supplementary Tables

| **Table 1 The hub genes detected by MCODE** | | | | |
| --- | --- | --- | --- | --- |
| **Cluster** | **Score (Density*Nodes)** | **Nodes** | **Edges** | **Node IDs** |
| **Yellow module** | | | | |
| 1 | 12.25 | 17 | 98 | OSR2, PEG3, EDNRA, CNRIP1, PDGFRB, NDN, HEYL, ZCCHC24, WT1-AS, GUCY1A2, TSHZ3, HAND2-AS1, DCN, CD248, COL6A2, JAM3, HIC1 |
| 2 | 3 | 3 | 3 | COL5A2, NID1, LDB2 |
| **Green module** | | | | |
| 1 | 7.429 | 15 | 52 | CDCA3, CCNB2, CCNB1, ARHGEF39, BUB1B, KIF2C, HJURP, NDC80, TACC3, AURKA, RAD54L, DLGAP5, NUSAP1, KIF15, CDC20 |
| 2 | 6.667 | 7 | 20 | NUF2, TTK, FANCI, CENPE, PRC1, UBE2C, CENPF |
| 3 | 3.8 | 11 | 19 | PIF1, NCAPG, CCNF, KIF18B, CCNA2, KIF20A, UHRF1, TOP2A, CDCA2, BIRC5, TPX2 |

| Table 2 Univariate and multivariate analysis of training set and testing set of TCGA database | | | | | | | | | | |
| --- | --- | --- | --- | --- | --- | --- | --- | --- | --- | --- |
|  | Univariate analysis | | |  | Multivariate analysis | | | | | |
| Variate | HR | 95%CI | *P* |  | HR | 95%CI | | *P* | |  |
| Testing set(n=271) |  |  |  |  |  |  |  |  |  |  |
| Age | 0.175 | 0.054-0.574 | 0.004005 |  | 0.315 |  | 0.089-1.111 |  | 0.07252 |  |
| Menopause status | 1.343 | 0.409-4.412 | 0.627238 |  | 0.299 |  | 0.070-1.276 |  | 0.10295 |  |
| BMI | 0.691 | 0.344-1.391 | 0.300725 |  | 1.027 |  | 0.499-2.114 |  | 0.94151 |  |
| Race | 1.003 | 0.481-2.089 | 0.993871 |  | 1.437 |  | 0.623-3.315 |  | 0.39474 |  |
| Histological type | 5.333 | 2.691-10.568 | 1.61E-06 |  | 1.820 |  | 0.759-4.365 |  | 0.17964 |  |
| Grade | 0.000 | 0 | 0.997 |  |  |  |  |  |  |  |
| FIGO stage | 3.567 | 1.804-7.053 | 0.000256 |  | 2.701 |  | 1.197-6.091 |  | 0.01667 |  |
| Risk score of the four genes | 3.525 | 1.951-6.368 | 2.99E-05 |  | 2.119 |  | 1.020-4.400 |  | 0.04394 |  |
|  |  |  |  |  |  |  |  |  |  |  |
| Training set(n=272) |  |  |  |  |  |  |  |  |  |  |
| Age | 0.512 | 0.244-1.076 | 0.077449 |  |  |  |  |  |  |  |
| Menopause status | 1.500 | 0.531-4.239 | 0.443992 |  |  |  |  |  |  |  |
| BMI | 0.947 | 0.498-1.800 | 0.868456 |  | 1.777 |  | 0.996-3.171 |  | 0.05166 |  |
| Race | 1.230 | 0.603-2.510 | 0.569573 |  | 1.421 |  | 0.741-2.728 |  | 0.29051 |  |
| Histological type | 3.283 | 1.771-6.084 | 0.000159 |  | 0.865 |  | 0.439-1.704 |  | 0.67441 |  |
| Grade | 0.075 | 0.010-0.547 | 0.010615 |  | 0.200 |  | 0.047-0.851 |  | 0.02935 |  |
| FIGO stage | 2.478 | 1.324-4.636 | 0.004535 |  | 3.866 |  | 2.156-6.929 |  | 1.00E-05 |  |
| Risk score of the four genes | 2.748 | 1.613-4.681 | 0.000201 |  | 1.801 |  | 1.035-3.133 |  | 0.03724 |  |
| HR, hazard ratio. CI: confidence interval. BMI, body mass index. FIGO: International Federation of Gynecology and Obstetrics | | | | | | | | | | |

| Table 3 GSEA enrichment results of hub genes | | | | | | | |
| --- | --- | --- | --- | --- | --- | --- | --- |
| Pathway | Core Enrichment | Size | Es | Nes | NOM P-val | FDR Q-val | Leading Edge |
| G2M checkpoint | TTK, AURKA, TPX2, KIF2C,  RAD54L, BIRC5, CCNF, KIF15, CCNA2, NDC80, UBE2C, CENPF, TOP2A, CENPE, CDC20, PRC1,  CCNB2 | 19 | 0.612 | 1.843 | 0.00331 | 0.00606 | tags=95%, list=60%, signal=128% |
| Mitotic spindle | TTK, AURKA, TPX2, KIF2C,  DLGAP5, BIRC5, KIF15,  NDC80, CENPF, TOP2A,  CENPE | 15 | 0.461 | 1.338 | 0.13127 | 0.13131 | tags=73%, list=45%, signal=86% |
|  | | | | | | | |

## Supplementary Figures


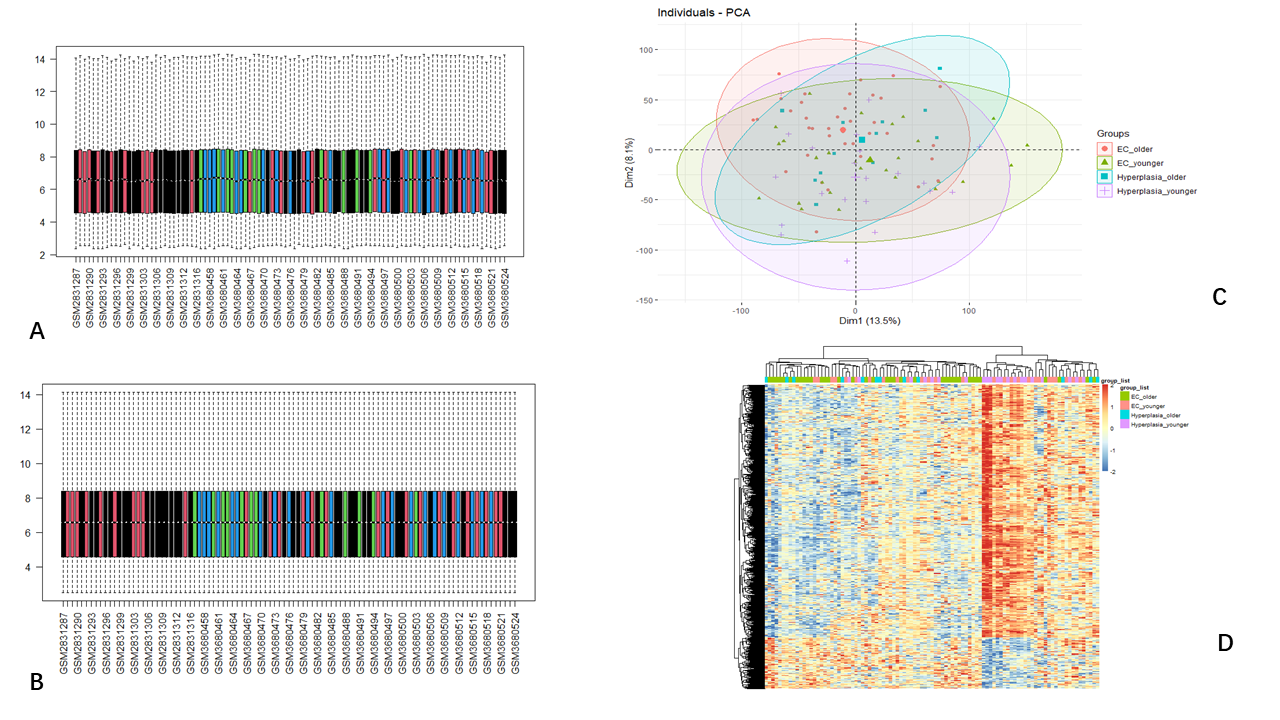


**Supplementary Figure 1.** Data process of gene expression in GES106191 datasets. (A)Boxplot of the original gene expression in GES106191 dataset and (B) after normalization. (C)PCA (Principal Component Analysis) plot. (D) Heatmap by groups.


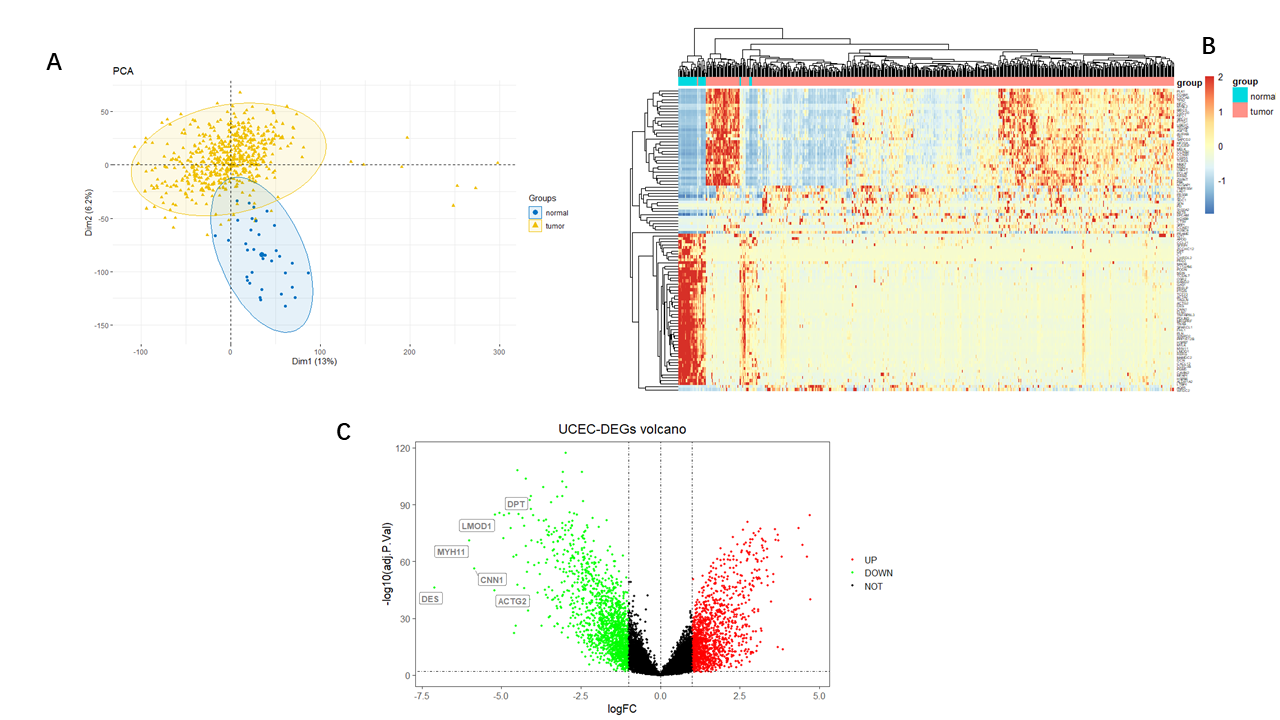


**Supplementary Figure 2.** Data process of gene expression in UCEC datasets of TCGA. (A) PCA plot of DEGs. (B)The heatmap of DEGs. (C) The volcano plot of DEGs.


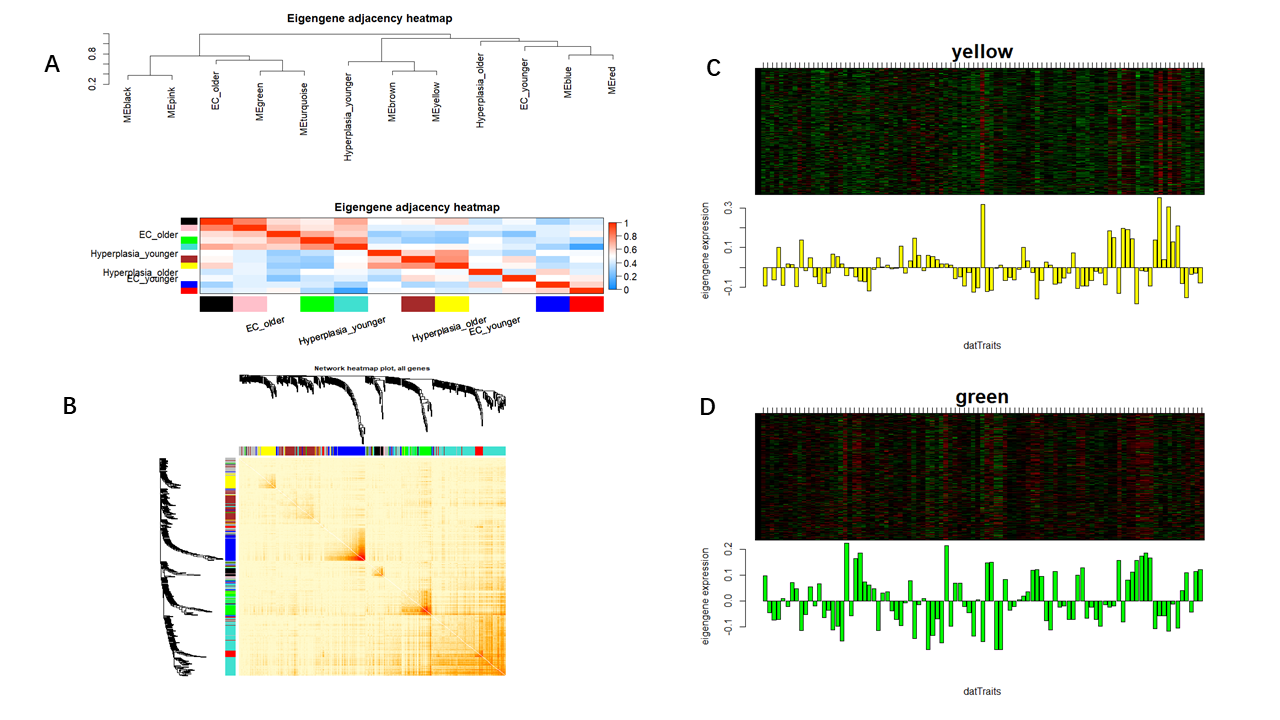


**Supplementary Figure 3.** The heatmaps of Construction of Weighted Gene Correlation Network Analysis. (A) Eigengene adjacency heatmap and dendrogram. (B) Network heatmap for all genes. (C) Yellow module heatmap and the eigengene expression. (D) Green module heatmap and the eigengene expression.


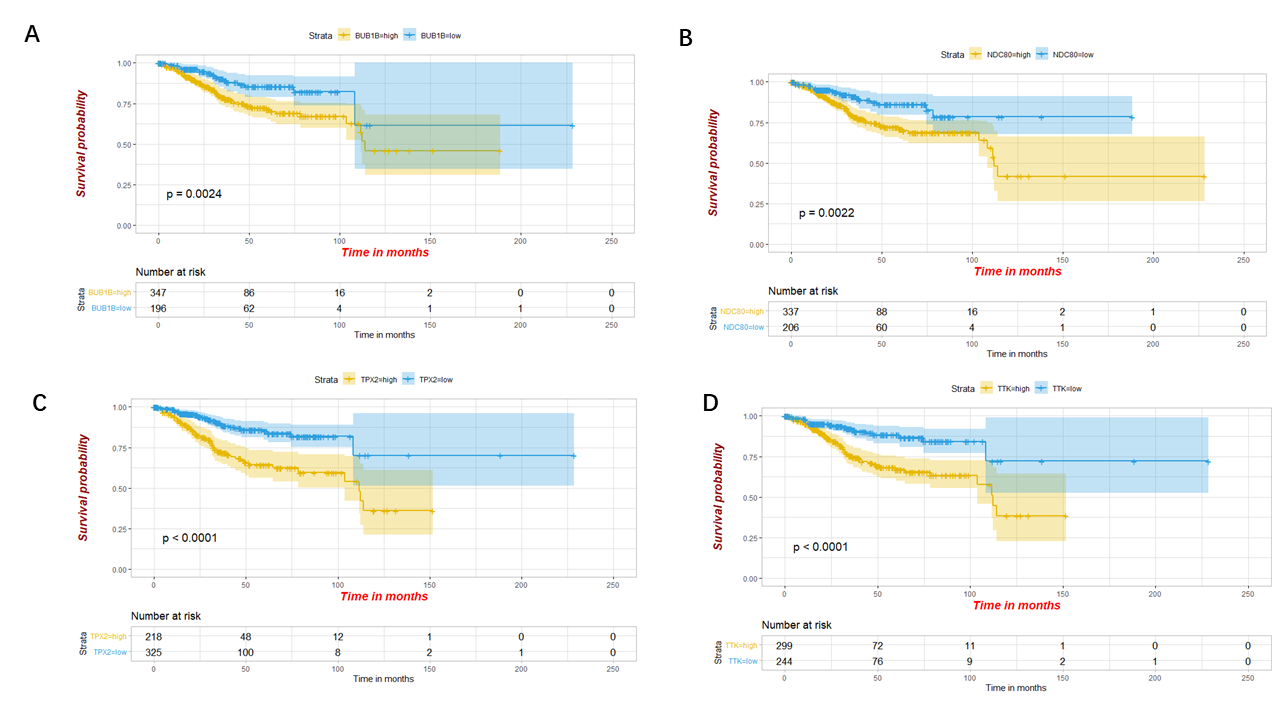


**Supplementary Figure 4.** The Kaplan-Meier plots of the 4 hub genes. (A) BUB1B; (B) NDC80; (C) TPX2; (D) TTK.


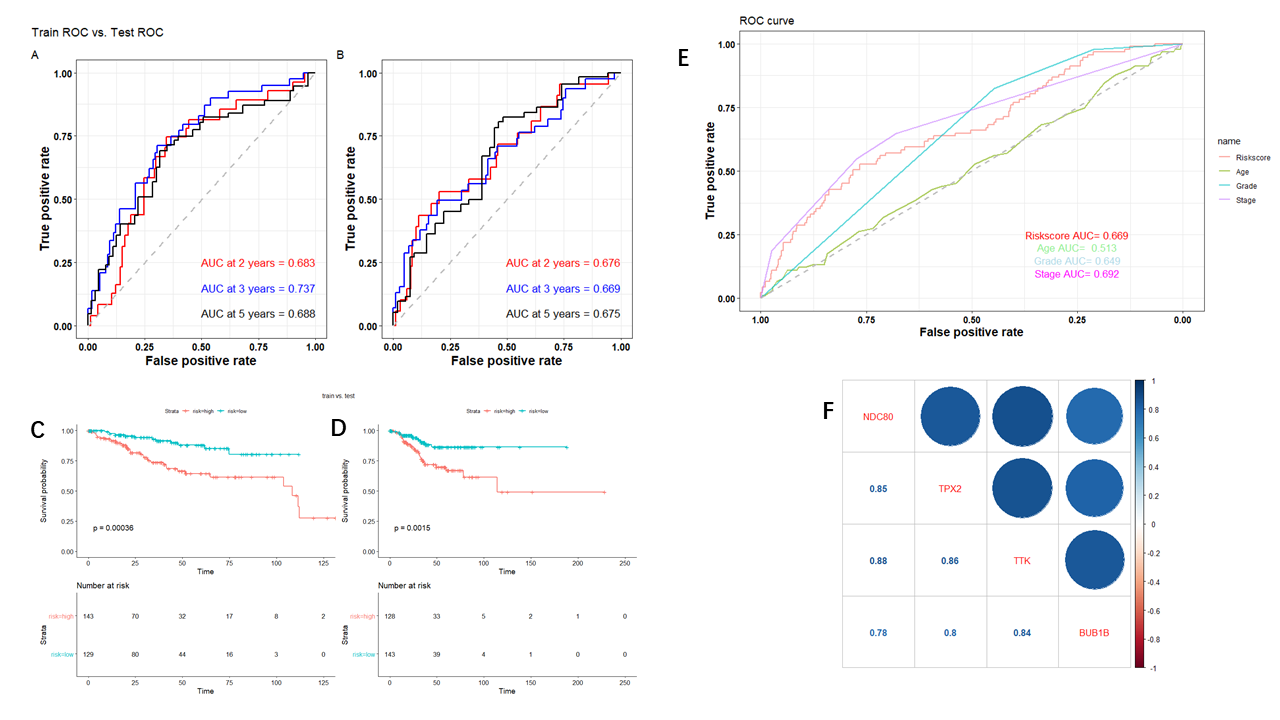


**Supplementary Figure 5.** ROC curves plots of the training set (A) and testing set (B). Kaplan Meier-plotter of overall survival of the four hub genes in Endometrial cancer (C and D). (E) ROC curves of the risk-score, age, grade and stage in prediction of overall survival for endometrial cancer. (F) Correlations of the four genes in UCEC dataset.


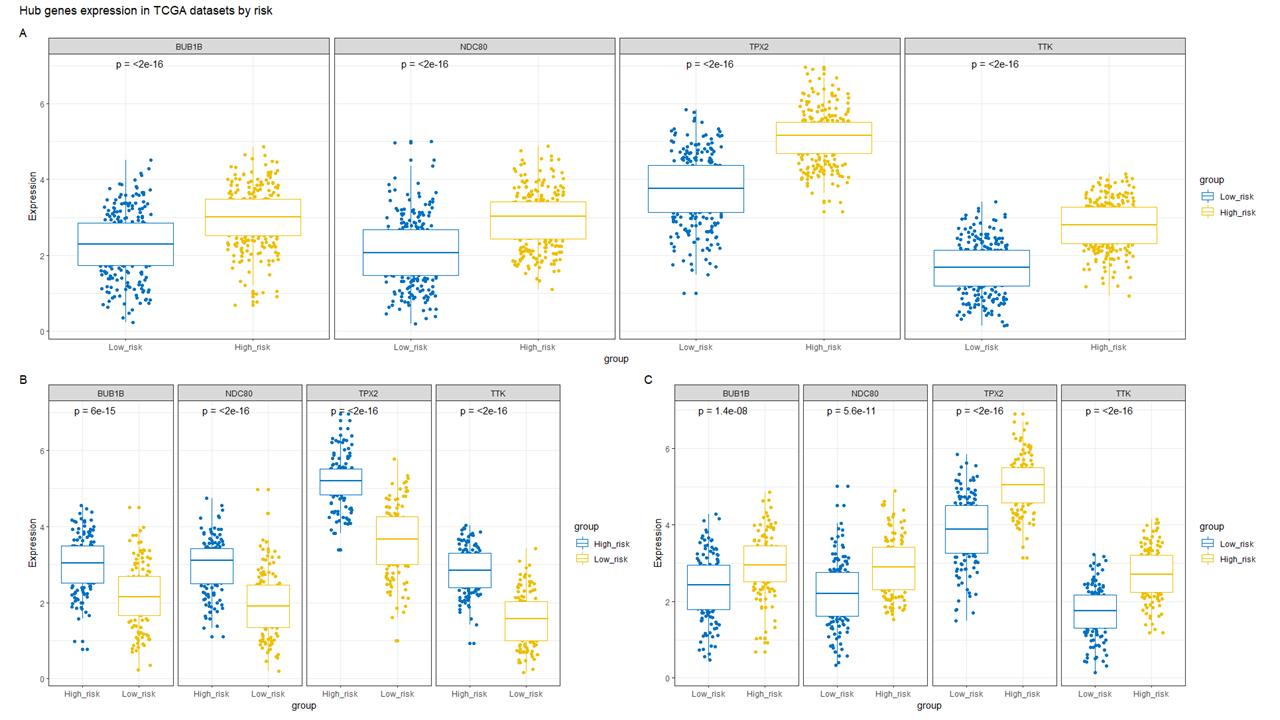


**Supplementary Figure 6.** Comparisons of the four hub genes expression in UCEC dataset in the whole dataset(A), training set (B), and testing set(C) between high-risk and low-risk group.
